# Lack of Associations between Elevated Serum Uric Acid and Components of Metabolic Syndrome Such as Hypertension, Dyslipidemia, and T2DM in Overweight and Obese Chinese Adults

**DOI:** 10.1155/2019/3175418

**Published:** 2019-12-04

**Authors:** Li Li, Qifa Song, Xi Yang

**Affiliations:** ^1^Department of Endocrinology and Metabolism, Ningbo First Hospital, Ningbo, Zhejiang, China; ^2^Ningbo Municipal Centre for Disease Control and Prevention, Ningbo, Zhejiang, China

## Abstract

The overweight and obese population experiences a higher occurrence of both hyperuricemia and metabolic syndrome. The present study was to explore the relationship between serum uric acid and metabolic syndrome-related risk factors among 409 obese Chinese adults (254 women and 155 men) with >24 kg/m^2^ BMI. Based on sex-specific reference ranges, 233 (57%) patients showed elevated serum uric acid. A total of 15 attributes were selected to assess the associations between elevated serum uric acid and components of metabolic syndrome, including serum uric acid, total cholesterol, HDL-C, LDL-C, triglyceride, systolic blood pressure, fasting blood glucose, glycosylated hemoglobin, HOMA-IR, alanine aminotransferase, creatinine, urine microalbumin, muscle mass amount, BMI, and age. Among the participants stratified into three groups of grade I, grade II, and grade III obesity, as well as among the participants stratified into male and female groups, univariate correlation analysis identified a negative association (*P* < 0.01) for age, positive associations (*P* < 0.01) for BMI, muscle mass, alanine aminotransferase, and creatinine. The stepwise multivariate logistic regression proved similar associations for age, BMI, creatinine, and alanine aminotransferase. No significant associations were testified between serum uric acid levels and cholesterol, HDL-C, LDL-C, triglyceride, fasting blood glucose, glycosylated hemoglobin, HOMA-IR, and urine microalbumin. Factor analysis illustrated that 15 attributes could be grouped into two common factors and five individual factors. A common underlying factor was identified among uric acid, muscle mass, creatinine, alanine aminotransferase, and BMI. The results indicate that serum uric acid has no apparent association with metabolic syndromes that are commonly characterized by hypertension, dyslipidemia, and T2DM.

## 1. Introduction

Overweight and obesity are chronic diseases with a manifestation of accumulating excessive fat mass in the body. People are generally considered to be obese when their body mass index (BMI) is over 30 kg/m^2^, while those with a BMI of 24–30 kg/m^2^ are defined as overweight [1]. This disorder often associates with numerous medical complications, mostly with metabolic syndrome [2] that is characteristic of type 2 diabetes mellitus (T2DM) [3], hypertension, dyslipidemia, and hyperuricemia [4]. These complications are considered to be risk factors for cardiovascular events. As one type of medical complication, hyperuricemia refers to the abnormally high level of uric acid in the blood. High concentrations of serum uric acid are associated with several medical disorders, such as gout and the formation of uric acid stones in the kidney [5]. Uric acid also has a proinflammatory effect, and its crystals can result in sudden pain in joints, an appearance of gout [6].

The relationship between elevated serum uric acid and metabolic syndrome has been reported to be inconsistent in previous researches. Several previous studies identified an association between elevated serum uric acid and components of metabolic syndrome such as cardiovascular events in obese people [2, 7], whereas some studies argued against such an association [4, 8]. It seems that patients at a particular hyperuricemia state, e.g., with uric acid calculi, showed an association with components of metabolic syndrome [2]. As hyperuricemia is prevalent among the overweight and obese population, whether hyperuricemia is related to metabolic syndrome or it is an independent factor among the whole overweight and obese population needs further investigation. This study was aimed at exploring the role of uric acid in metabolic syndrome. The associations between serum uric acid and the attributes that reflected liver and kidney impairment, cardiovascular risk, and impaired glucose tolerance were analyzed. We conducted univariate analysis and multivariate logistic regression analysis. We also conducted factor analysis to determine the underlying common factor among the multidimensional risk factors for metabolic syndrome.

## 2. Material and Methods

### 2.1. Study Design and Study Population

This study was approved by the Ethics Committee of Ningbo First Hospital and followed the Declaration of Helsinki. Written consent was obtained from all participants. The study consisted of the following steps, including participant enrollment, attribute selection and measurement, univariate correlation analysis and multivariate generalized linear model regression analysis, factor analysis, and final professional interpretation. Participants with BMI ≥ 24 kg/m^2^ were 18–75 years old and sought weight loss therapy in the hospital from January 2015 to December 2018. A physical examination was performed on the participants, including measurement of weight, height, and systolic blood pressure. Data from the patients were retrospectively analyzed.

### 2.2. Definition and Measurement of Attribute Values

Based on professional knowledge and previous literature, 15 attributes were selected, including total cholesterol, high-density lipoprotein cholesterol(HDL-C), low-density lipoprotein cholesterol(LDL-C), and triglyceride representing dyslipidemia; LDL-C and systolic blood pressure that were used as risk factors for cardiovascular events; fasting blood glucose, glycosylated hemoglobin (HbA1c), and homeostatic model assessment index of insulin resistance (HOMA-IR) that were used as indices for impaired glucose tolerance and T2DM; alanine aminotransferase as an index for liver impairment, as well as elevated creatinine and urine microalbumin as indices for renal impairment [9]; and BMI and age (Table 1).

After at least 12 h overnight fasting, venous blood samples were collected for measurements of uric acid, creatinine, alanine aminotransferase, total cholesterol, HDL-C, LDL-C, triglyceride, fasting blood glucose, and HbA1c. Serum uric acid was determined by enzymatic spectrophotometry. Hyperuricemia was defined as the serum uric acid level exceeding 416 *μ*mol/L (7 mg/dL) for men and 357 *μ*mol/L (6 mg/dL) for women [10]. HOMA-IR calculated as fasting plasma glucose (mmol/L) × fasting insulin (mIU/L)/22.5 was used to estimate insulin resistance [11]. A random urine sample was obtained to measure creatinine and microalbumin concentration. Microalbumin secretion in the urine was expressed in urinary albumin/creatinine ratio (in milligrams albumin per gram creatinine) [12]. Fat-free mass, which was expressed in muscle mass amount (in kg) from sex-specific equations based on total body water, was estimated via bioelectric impedance analysis (GAIA KIKA, Jawon company, Korea) [13].

### 2.3. Statistical Analysis

All statistics were derived by R software that is an open-source programming language and has plenty of libraries of statistical packages and graphic tools [14, 15]. Significance for all statistical results was determined by 95% confidence interval or *P* < 0.05. Because numerous factors that were related to metabolic syndrome and some anthropological indices such as sex and age might affect uric acid levels, these factors might cause confounding effects in the analysis of associations. The univariate correlation analysis was conducted with sex and BMI as stratification factors to reduce the confounding effect. A multivariate regression analysis was used to remove uncertain confounding effects and prove the correlation obtained by the univariate analysis. The associations between serum uric acid and individual variables were reviewed to specify probable associative factors for the subsequent multivariate logistic regression. Uric acid values were transformed into binomial variables of 0 or 1 according to sex-specific reference ranges and were thereby used as the dependent variable for multivariate logistic regression. Finally, all attributes were evaluated by factor analysis to detect their underlying common factors. The factor analysis would shed light on the question of whether these attributes were independent factors or connected by a common linking factor.

## 3. Results

Overall, 409 participants (254 women and 155 men) with >24 kg/m^2^ BMI were enrolled. Based on sex-specific reference ranges, 233 (57%) exhibited an elevated uric acid level. The associations between serum uric acid and every attribute among all participants were firstly investigated. A negative association (*P* < 0.01) was found between serum uric acid and age, whereas positive associations (*P* < 0.01) were found between serum uric acid and BMI, muscle mass, alanine aminotransferase, creatinine, systolic blood pressure, and HOMA-IR (Table 1). Weak associations (0.01 < *P* < 0.05) were observed for total cholesterol and HDL-C. No associations (*P* > 0.05) were observed between serum uric acid and urine microalbumin, triglyceride, fasting blood glucose, glycosylated hemoglobin, and LDL-C.

Then, the study participants were stratified by three ranges of BMI values representing grade I, grade II, and grade III obesity (Table 2). Compared with the resultant associations among the whole study population, only negative association (*P* < 0.01) for age and positive associations (*P* < 0.01) for muscle mass, alanine aminotransferase, and creatinine were still present. When the participants were stratified into male and female groups, only age, BMI, creatinine, muscle mass, and alanine aminotransferase were significantly associative (*P* < 0.05) (Table 3). The results showed that these associations that remained in the stratified groups were independent of the stratification factors such as weight and sex.

**Figure 1:**
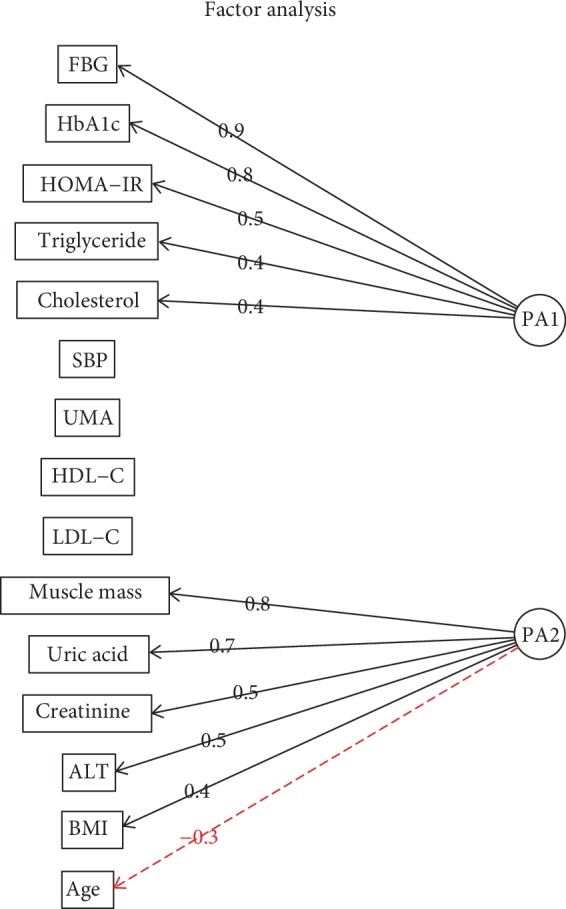
Factor analysis of 15 attributes. There are two common underlying factors (PA1 and PA2) and five independent factors. Abbreviations: see Table 1.

To further assess the associative levels and reduce confounding effects from multivariate attributes, we designated normal and abnormal uric acid as 0 and 1 according to sex-specific reference ranges and performed stepwise multivariate logistic regressions (Table 4). Age, BMI, creatinine, and alanine aminotransferase were tested to be significantly associated with uric acid. These multivariate results were identical to the findings from the stratification method by sex. Additionally, factor analysis was carried out to explore the common factor that existed as an underlying link among all attributes. The results illustrated that the attributes could be grouped into two common factors PA1 and PA2, as well as five individual factors, including age, HDL-C, LDL-C, urine microalbumin, and systolic blood pressure (Figure 1). PA1 that contained fasting blood glucose, glycosylated hemoglobin, HOMA-IR, cholesterol, and triglyceride was independent of uric acid. PA2 consisted of uric acid, muscle mass, creatinine, alanine aminotransferase, and BMI, indicating a common link among the components of PA2 that included uric acid.

As a final interpretation, there was a negative association between serum uric acid and age (Table 4). The positive associations were present for BMI, creatinine, muscle mass, and alanine aminotransferase. Serum uric acid levels were independent of cholesterol, HDL-C, LDL-C, triglyceride, fasting blood glucose, glycosylated hemoglobin, HOMA-IR, and urine microalbumin. There was a common underlying factor for uric acid, muscle mass, creatinine, alanine aminotransferase, and BMI.

## 4. Discussion

In public, uric acid has become a popular topic of a healthy lifestyle. In the academic community, serum uric acid is also a continuing issue as to whether or not it is related to metabolic syndrome. Especially among the overweight and obese population, they experience a higher occurrence of both hyperuricemia and metabolic syndrome [4]. Our findings have shown that uric acid is significantly associated only with age, BMI, creatinine, and alanine aminotransferase, and it is not associated with HDL-C, LDL-C, fasting blood glucose, HbA1c, HOMA-IR, and urine microalbumin. These results safely indicate that uric acid has very limited association with components of metabolic syndrome that is commonly characterized by hypertension, dyslipidemia, and T2DM. The interesting finding was that the younger people became a more focused target of elevated uric acid (Table 1). This phenomenon needs further investigation.

In the past decades, several studies have assessed the relationship between serum uric acid and components of metabolic syndrome in different populations. It is commonly believed that an association between elevated serum uric acid and obesity is present [16]. As one of the leading clinical outcomes of obesity is metabolic syndrome, the main uncertainty is whether serum uric acid can affect health concurrently with metabolic risk factors, in other words, whether it is also a risk factor for metabolic syndrome. Nevertheless, answers to this issue seem to be inconsistent in previous researches. Some studies indicated an association between hyperuricemia and a number of metabolic diseases, including diabetes mellitus [17], dyslipidemia, hypertension [18], and even cardiovascular diseases and renal function decline [19]. In contrast, there were researches that indicated a lack of such an association and identified elevated serum uric acid as an independent factor [4, 8, 20]. Among patients with cardiovascular diseases and hypertension, hyperuricemia can also be a result of the dysfunction of multiorgans instead of a cause due to reduced excretion of urinary uric acid.

Although many factors are metabolically relevant, those pertinent to lipid, glucose, and blood pressure are most notable. We selected several measurements that were considered to be risk factors for the metabolic syndrome and were usually analyzed in previous researches to explore their associations with serum uric acid. These markers included elevated HOMA-IR, HbA1c, and fasting blood glucose that indicated impaired glucose metabolism; elevated LDL-C and systolic blood pressure that were risk factors for cardiovascular events; total cholesterol, HDL-C, LDL-C, and triglyceride that were markers of dyslipidemia; elevated alanine aminotransferase for liver impairment; and elevated creatinine and urine microalbumin for renal impairment. In deciphering the web of associations among numerous attributes, another noteworthy challenge is the confounding factors. Professionally, age, BMI, and sex are among the most important confounding factors for serum uric acid. As in the study, discrepancy existed between associations among the whole study population and subpopulations by BMI and sex (Tables 1–4). This discrepancy necessitated professional considerations in analyzing and interpreting the associations.

Our results revealed no associations with classical lipid metabolism characterized by elevated LDL-C and triglyceride, as well as no association with hypertension. These attributes are the major risk factors for cardiovascular diseases. The results are contrasting with some previous studies that argued for a significant association between elevated uric acid levels and cardiovascular illnesses [20]. Similar to our results, such an association was not observed in Chinese T2DM patients [4]. As to glucose metabolism, the present study failed to find apparent associations likewise for fasting blood glucose, glycosylated hemoglobin, and HOMA-IR, which was distinct from previous literature that proved strong associations between T2DM and hyperuricemia [21]. In terms of urine microalbumin as an index of renal impairment, the present study also observed no significant association.

There were positive associations for BMI, creatinine, muscle mass, and alanine aminotransferase, as well as a negative association for age. It seemed that sex was a confounding factor for creatinine, muscle mass, and alanine aminotransferase as sex-stratified subgroups showed reduced correlation strength. Nevertheless, the positive associations were still present in the subgroups. Factor analysis revealed that all the variables with positive associations and uric acid belonged to a common underlying factor (Figure 1). Factor analysis is a statistical method used to describe variability among observed, correlated variables to find a potentially less number of unobserved variables that are called factors. For example, some observed variables may alter their values following a common underlying factor. Factor analysis can further uncover whether the variables are interdependent or link to each other. In the present study, factor analysis also proved that uric acid was not significantly associated with common risk factors for metabolic syndrome. This array of concurrently existed factors seemed to be related to the muscle mass and body mass. We hypothesized that an increased amount of body mass means more cell amount that needs more structural materials such as nucleic acids and proteins, and consequently leads to increased uric acid production. However, this increased body bulk has little association with metabolic syndrome. One of the exceptions was the association with alanine aminotransferase, which was previously observed between uric acid and nonalcoholic fatty liver disease [22], which might result from obesity.

Different from other metabolites such as sugars, lipids, and some intermediate products of proteins, the authentic role of uric acid and its influence on human health are always intricate. Uric acid, especially its crystals, can exert inflammatory effects on tissues and cause gout and renal stones. However, in humans and many primates, uric acid is the end product of purine metabolism and is mainly excreted in the urine, whereas in most other mammals, it is further oxidized to allantoin by the enzyme uricase [23]. The loss of uricase in higher primates parallels the similar loss of the ability to synthesize ascorbic acid that is an effective antioxidant, leading to the hypothesis that urate, the salt of uric acid, may take on the ascorbates' antioxidant role in primates [24]. Consequently, hydrogen urate ion is considered to account for over half of the antioxidant capacity of plasma [25]. From this theoretical basis, the role of uric acid is not definitely good or bad for human health. This theory supports our findings that there are no apparent associations between serum uric acid and most of the metabolic syndrome-causative risk factors.

## 5. Conclusion

Serum uric acid is inversely related to age and positively related to creatinine, BMI, and alanine aminotransferase. The present study illustrates that serum uric acid is not a synergistic factor for the components of metabolic syndrome. No significant associations are present between serum uric acid and dyslipidemia, T2DM, cardiovascular events, kidney impairment, and hypertension.

## Figures and Tables

**Table 1:** Correlations between serum uric acid and attributes among 409 participants.

Attribute	Uric acid, *n* = 409							Interpretation of correlation
	Mean	SD	Min	Max	*r*	95% CI	*P*	
Uric acid (*μ*mol/L)	407	100	205	758	—	—	—	—
Age (y)	31.0	9.2	18	71	-0.33	-0.41–0.24	<0.01	Negative
BMI (kg/m^2^)	33.4	5.1	24.6	55.1	0.30	0.21–0.39	<0.01	Positive
Muscle mass (kg)	53.6	9.8	30.2	90.0	0.45	0.37–0.52	<0.01	Positive
SBP (mmHg)	132	14.4	100	188	0.14	0.05–0.24	<0.01	Positive
ALT (IU/L)	58.1	48.7	5	258	0.40	0.31–0.48	<0.01	Positive
Creatinine (*μ*mol/L)	62.0	14.0	33	114	0.47	0.39–0.55	<0.01	Positive
Cholesterol (mmol/L)	5.2	1.0	2.39	12.1	0.10	0.01–0.20	0.04	Weakly positive
HDL-C (mmol/L)	1.6	1.0	0.61	7.20	0.11	0.01–0.20	0.03	Weakly positive
LDL-C (mmol/L)	2.8	1.0	0.53	5.49	-0.04	—	0.38	—
Triglyceride (mmol/L)	1.90	1.5	0.42	23.5	0.04	—	0.44	—
FBG (mmol/L)	5.8	1.5	3.72	17.7	0.04	—	0.38	—
HbA1c (%)	5.7	1.0	4.4	12.6	0.05	—	0.45	—
HOMA-IR	7.2	5.3	0.71	43.50	0.19	0.09–0.28	<0.01	Positive
UMA (mg/g creatinine)	6.7	15.8	0.21	205.0	0.09	—	0.06	—

Abbreviations: CI: confidence interval; SD: standard deviation; SBP: systolic blood pressure; ALT: alanine aminotransferase; HDL-C: HDL cholesterol; LDL-C: LDL cholesterol; FBG: fasting blood glucose; HOMA-IR: homeostatic model assessment index of insulin resistance; UMA: urine microalbumin.

**Table 2:** Correlations between serum uric acid and attributes among participants stratified by BMI.

Attribute	Grade I BMI (24–29.9), *n* = 108					Grade II BMI (30–34.9), *n* = 187					Grade III and over BMI (35–), *n* = 134					Interpretation of correlation
	Mean	SD	*r*	95% CI	*P*	Mean	SD	*r*	95% CI	*P*	Mean	SD	*r*	95% CI	*P*	
Uric acid	369	92	—	—	—	405	92	—	—	—	442	128	—	—	—	
Age	31.5	9.2	-0.28	-0.45–0.10	<0.01	30.8	9.1	-0.28	-0.41– -0.13	<0.01	30.9	9.6	-0.43	-0.57– -0.28	<0.01	Negative
Muscle mass	47.6	7.0	0.34	0.16–0.50	<0.01	53.4	8.1	0.42	0.29–0.54	<0.01	59.1	10.8	0.37	0.21–0.51	<0.01	Positive
SBP	128.1	14.8	0.20	0.10–0.37	0.04	130.6	13.2	0.11	—	0.14	136.5	14.4	-0.02	—	0.76	—
ALT	37.1	30.6	0.41	0.24–0.56	<0.01	58.3	49.1	0.22	0.07–0.35	<0.01	75.5	53.9	0.46	0.31–0.59	<0.01	Positive
Creatinine	60.0	13.6	0.53	0.38–0.66	<0.01	62.6	13.7	0.47	0.35–0.58	<0.01	63.0	15.1	0.43	0.28–0.56	<0.01	Positive
Cholesterol	5.1	5.1	-0.05	—	0.57	5.2	0.9	0.07	—	0.38	5.3	1.0	0.20	0.03–0.36	0.02	—
HDL-C	1.5	0.8	-0.14	—	0.16	1.7	1.1	0.04	—	0.56	1.6	1.1	0.15	—	0.09	—
LDL-C	2.9	0.9	-0.20	—	0.18	2.8	1.1	-0.02	—	0.75	2.9	1.1	0.01	—	0.97	—
Triglyceride	1.8	2.2	0.05	—	0.78	2.2	1.4	0.19	0.05–0.33	0.01	1.9	1.0	0.13	—	0.13	—
FBG	5.6	1.7	0.05	—	0.64	5.8	1.4	0.07	—	0.34	6.0	1.4	-0.07	—	0.42	—
HbA1c	5.5	1.0	0.04	—	0.70	5.7	1.0	0.04	—	0.40	5.9	1.0	0.02	—	0.41	—
HOMA-IR	5.3	3.5	0.23	0.04–0.44	0.02	6.6	4.6	0.09	—	0.21	9.70	6.3	0.08	—	0.37	—
UMA	3.7	5.1	0.05	—	0.61	4.9	8.0	0.05	—	0.49	11.7	25.6	0.05	—	0.6	—

Abbreviations and units of attributes: see Table 1 legend.

**Table 3:** Correlations between serum uric acid and attributes among participants stratified by sex.

Attribute	Male, *n* = 155					Female, *n* = 254					Interpretation of correlation
	Mean	SD	*r*	95% CI	*P*	Mean	SD	*r*	95% CI	*P*	
Uric acid	470	97	—	—	—	367	82	—	—	—	—
Age	29.7	8.3	-0.27	-0.42–0.13	<0.01	31.8	9.7	-0.34	-0.44– -0.19	<0.01	Negative
BMI	34.4	4.8	0.31	0.17–0.45	<0.01	32.8	5.1	0.23	0.10–0.34	<0.01	Positive
Muscle mass	61.7	9.3	0.21	0.04–0.35	0.01	48.7	6.15	0.20	0.06–0.32	<0.01	Positive
SBP	134.5	15.0	0.04	—	0.55	130.1	13.8	0.07	—	0.55	—
ALT	83.9	55.6	0.18	0.02–0.33	0.03	42.4	35.9	0.32	0.03–0.32	0.03	Positive
Creatinine	74.3	12.3	0.24	0.08–0.38	<0.01	54.3	9.0	0.21	0.06–0.36	<0.01	Positive
Cholesterol	5.3	1.1	0.09	—	0.31	5.2	0.9	0.10	—	0.31	—
HDL-C	1.5	1.1	0.14	—	0.11	1.7	1.0	0.18	—	0.11	—
LDL-C	3.0	1.0	-0.03	—	0.77	2.8	1.0	-0.12	—	0.77	—
Triglyceride	2.2	2.2	-0.07	—	0.78	1.8	1.0	0.16	0.04–0.28	0.01	—
FBG	6.0	1.8	-0.11	—	0.23	5.7	1.3	0.09	—	0.23	—
HbA1c	5.9	1.0	-0.15	—	0.88	5.6	1.0	0.16	—	0.80	—
HOMA-IR	7.7	5.0	0.05	—	0.53	6.9	5.4	0.25	—	0.53	—
UMA	9.0	22.7	0.03	—	0.71	5.3	9.1	0.07	—	0.26	—

Abbreviations and units of attributes: see Table 1 legend.

**Table 4:** Summary of univariate correlation and multivariate logistic regression results and factor analysis of attributes.

Attribute	Univariate correlation analysis			Multivariate logistic regression	Factor analysis	Final interpretation of associations
	Whole population	Stratification by BMI	Stratification by sex			
Age	Negative	Negative	Negative	Negative	PA2^∗^	Negative
BMI	Positive	—	Positive	Positive	PA2	Positive
Muscle mass	Positive	Positive	Positive	*P* = 0.09	PA2	Positive
SBP	—	*P* = 0.04	—	—	Individual	—
ALT	Positive	Positive	Positive	Positive	PA2	Positive
Creatinine	Positive	Positive	Positive	Positive	PA2	Positive
Cholesterol	*P* = 0.04	—	—	—	PA1	—
HDL-C	*P* = 0.03	—	—	—	Individual	—
LDL-C	—	—	—	—	Individual	—
Triglyceride	—	—	—	—	PA1	—
FBG	—	—	—	—	PA1	—
HbA1c	—	—	—	—	PA1	—
HOMA-IR	Positive	—	—	—	PA1	—
UMA	—	—	—	—	Individual	—

Note: abbreviations and units of attributes: see Table 1 legend. ^∗^PA: principal axis factor analysis. Negative and positive associations are associations with *P* < 0.01.

## Data Availability

All data used in this study can be obtained from the corresponding author via email address.

## Abbreviations

BMI: Body mass index
T2DM: Type 2 diabetes mellitus
HDL-C: High-density lipoprotein cholesterol
LDL-C: Low-density lipoprotein cholesterol
HOMA-IR: Homeostatic model assessment index of insulin resistance.
